# Energy metabolism in osteoprogenitors and osteoblasts: Role of the pentose phosphate pathway

**DOI:** 10.1016/j.jbc.2024.108016

**Published:** 2024-11-26

**Authors:** Sarah E. Catheline, Charles O. Smith, Matthew McArthur, Chen Yu, Paul S. Brookes, Roman A. Eliseev

**Affiliations:** 1 Center for Musculoskeletal Research, University of Rochester, Rochester, New York, USA; 2 Department of Anesthesiology and Perioperative Medicine, University of Rochester, Rochester, New York, USA; 3 Department of Pharmacology & Physiology, University of Rochester, Rochester, New York, USA; 4 Department of Pathology, University of Rochester, Rochester, New York, USA

**Keywords:** osteoprogenitors, osteoblasts, pentose phosphate pathway, mitochondria, malate-aspartate shuttle

## Abstract

Bioenergetic preferences of osteolineage cells, including osteoprogenitors and osteoblasts (OBs), are a matter of intense debate. Early studies pointed to OB reliance on glucose and aerobic glycolysis while more recent works indicated the importance of glutamine as a mitochondrial fuel. Aiming to clarify this issue, we performed metabolic tracing of ^13^C-labeled glucose and glutamine in human osteolineage cells: bone marrow stromal (a.k.a. mesenchymal stem) cells and bone marrow stromal cell-derived OBs. Glucose tracing showed noncanonical direction of glucose metabolism with high labeling of early glycolytic steps and the pentose phosphate pathway (PPP) but very low labeling of late glycolytic steps and the Krebs cycle. Labeling of Krebs cycle and late steps of glycolysis was primarily from glutamine. These data suggest that in osteolineage cells, glucose is metabolized primarily *via* the PPP while glutamine is metabolized in the mitochondria, also feeding into the late steps of glycolysis likely *via* the malate-aspartate shuttle. This metabolic setup did not change after induction of differentiation. To evaluate the importance of this setup for osteolineage cells, we used the inhibitors of either PPP or malate-aspartate shuttle and observed a significant reduction in both cell growth and ability to differentiate. In sum, we observed a distinct metabolic wiring in osteolineage cells with high flux of glucose through the PPP and glutamine flux fueling both mitochondria and late steps of glycolysis. This wiring likely reflects their unique capacity to rapidly proliferate and produce extracellular matrix, *e.g.*, after bone fracture.

Cells produce energy to fuel their bioenergetic needs using the cytosolic process of glycolysis that metabolizes glucose into pyruvate. Pyruvate can then be processed one of two ways, into acetyl-CoA in mitochondria or into lactate in the cytosol. Glycolysis is indelibly linked to another cytosolic pathway known as the pentose phosphate pathway (PPP), as once glucose has been converted into glucose-6-phosphate, it can be either converted to fructose-6-phosphate and continue with glycolysis, or it can be reduced by glucose-6-phosphate dehydrogenase to produce NADPH and 6-phosphogluconolactone and continue down the PPP. By producing NADPH, PPP allows for redox reactions and biosynthesis to occur, which facilitates DNA/RNA synthesis during proliferation (1, 2). The production of NADPH also allows for reduction of oxidized moieties abundantly present on precursors of functional proteins. One example is proline shown to be a necessary molecule in the formation of extracellular matrix proteins important for osteoblasts (OBs) such as Osterix and Collagen I (3, 4). Despite this linkage, the role of the PPP has remained largely unexplored in many tissue types, including bone.

While much is known about OB differentiation and their role in maintaining bone homeostasis by producing extracellular matrix and mineralizing it, little is settled about the bioenergetic needs of this cell type (5). Early studies strictly considered glucose as the only potential source of energy fueling OBs without considering that alternative sources such as fatty acids or glutamine may also play a role (6, 7). The importance of glycolysis for OBs and osteoprogenitors has been since confirmed by numerous studies including ours that showed deleterious effects of glycolysis inhibitors on OB differentiation and function (8, 9, 10). However, even more robust literature has been accumulated recently on the importance of mitochondrial metabolism for OB differentiation and function (8, 11, 12, 13, 14). Our point is that these processes do not have to be mutually exclusive and we just need to study it holistically and in connection to each other. Also, most of these recent studies focused on either glycolysis or mitochondria but not any alternative pathways, such as the PPP (8, 11, 12, 13, 14, 15, 16, 17, 18, 19, 20, 21).

Given the knowledge that bone formation becomes deficient during the aging process, better understanding how the OB prefers to utilize metabolic pathways could allow for leveraging of these pathways to improve OB function during aging (5, 22). We and others have previously shown that OB dysfunction in the aged context can be caused by enhanced oxidative stress and changes to their metabolic programming to shift toward glycolysis and away from oxidative phosphorylation and the PPP (13, 23). This glycolytic shift can be reversed by shunting glucose away from lactate production by administering a systemic lactate dehydrogenase A inhibitor, oxamate (10).

In this study, we aim to answer lingering questions regarding metabolic flux during the process of differentiation of bone marrow stromal (a.k.a. mesenchymal stem) cells (BMSCs) down the osteogenic lineage using ^13^C_6_-glucose or ^13^C_5_-glutamine. We also begin to test the hypothesis that the PPP may be essentially contributing to bone homeostasis by maintaining production of NADPH and allowing for critical biosynthesis of proline that facilitates bone extracellular matrix protein production. Overall, our findings suggest the importance of PPP and malate-aspartate shuttle (MAS) as their inhibition can blunt osteogenic differentiation of BMSCs into OBs and affect their proliferative capacity.

## Results

### Metabolic tracing of BMSCs displays preferential use of PPP and glutamine metabolism during osteogenic differentiation

**Figure 1:**
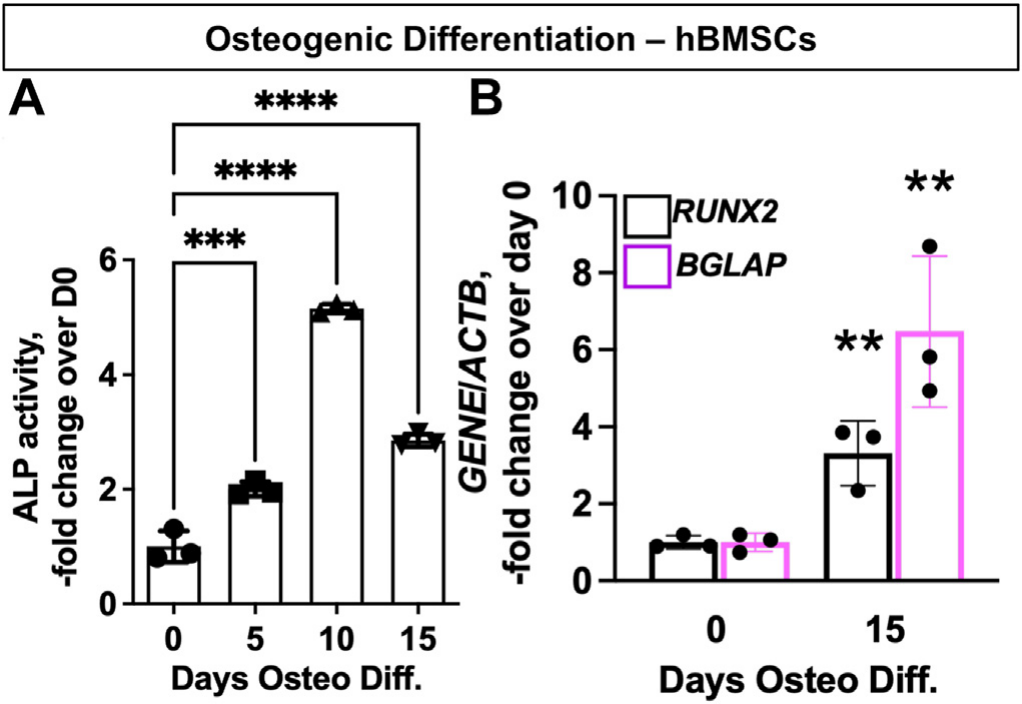
**Time course of differentiation of hBMSCs down the osteogenic lineage.** hBMSCs were cultured in osteogenic media for 0, 5, 10, and 15 days. *A*, alkaline phosphatase, ALP, activity was measured and normalized to protein content of each sample. *B*, osteogenic gene expression was measured using real-time RT-qPCR; gene of interest expression level was normalized to β-actin expression and day 0 samples to calculate fold change. Data are mean ± SD (n = 3 biological replicates). Here and in other figures, *p* value is determined *via* either unpaired *t* test or one-way ANOVA with *post hoc* Tukey multiple comparisons test. ∗∗*p* < 0.01; ∗∗∗*p* < 0.001; ∗∗∗∗*p* < 0.0001. BMSC, bone marrow stromal (a.k.a. mesenchymal stem) cells; hBMSCs, human BMSCs.

In order to elucidate the metabolic pathways utilized by osteogenic lineage cells and their precursors, ^13^C-glucose or ^13^C-glutamine were used in conjunction with metabolomics performed with liquid chromatography-mass spectroscopy. Primary human BMSCs (hBMSCs) were osteogenically differentiated for 0, 5, 10, or 15 days, and differentiation progress was tracked using alkaline phosphatase (ALP) activity and gene expression of osteogenic markers. ALP activity was progressively enhanced starting at day 5 and peaking at day 10 of differentiation, while gene expression of *RUNX2*, a critical transcription factor during OB differentiation, as well as the mature osteolineage marker *BGLAP* were both found to be upregulated at day 15 (Fig. 1, *A* and *B*). Our previous experiments have shown that mineralization and late OB markers are upregulated at 2 weeks of osteoinduction, and we used this time point to evaluate effects on osteoinduction in subsequent experiments (data not shown).

**Figure 2:**
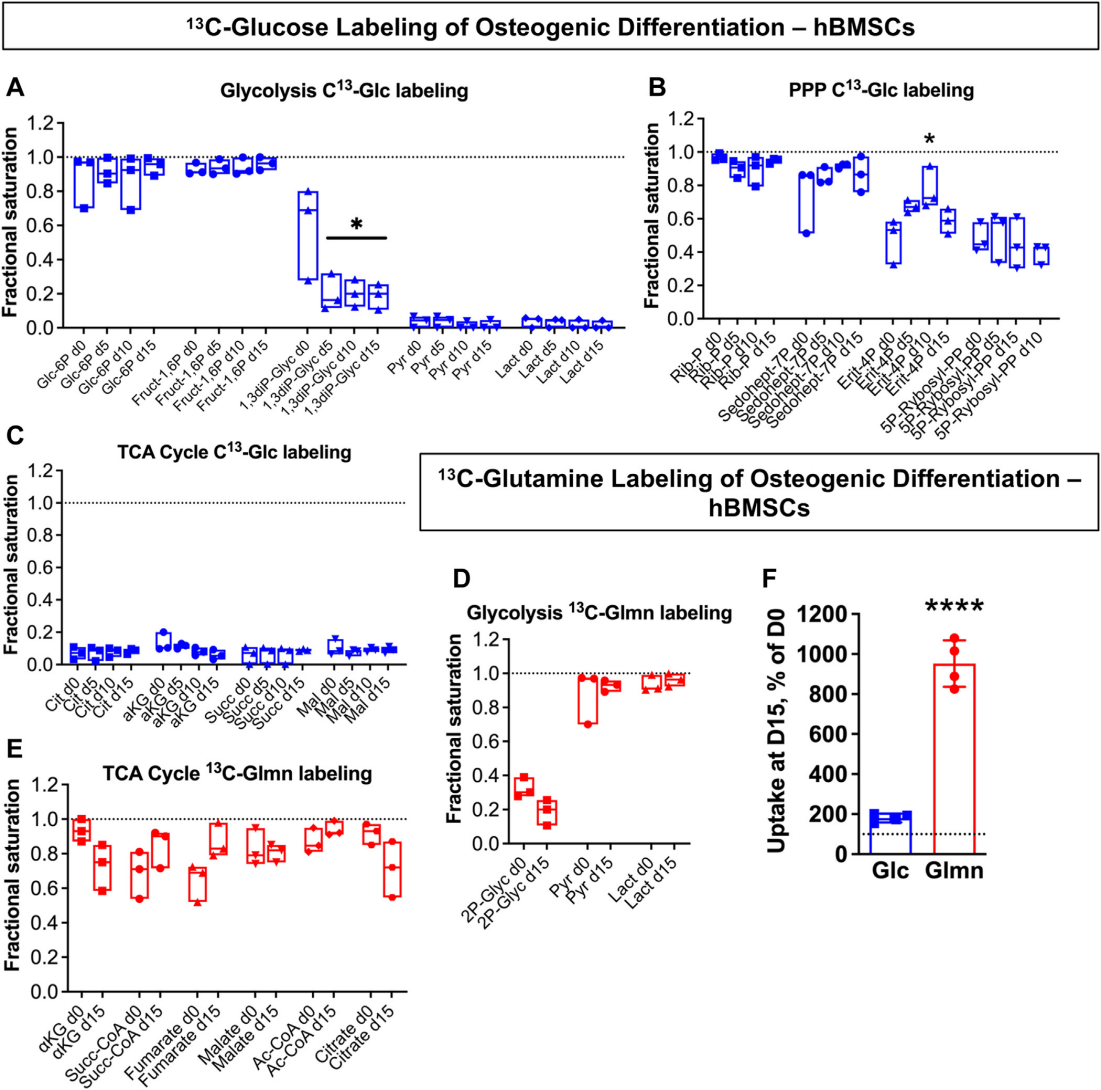
**Metabolic tracing shows a preferential flow of glucose toward the pentose phosphate pathway and enhanced glutamine uptake in osteogenically differentiated BMSCs.***A*–*E*, hBMSCs were cultured in osteogenic media for 0, 5, 10, or 15 days and subsequently incubated with either 5 mM ^13^C_6_ glucose or 1 mM ^13^C_5_ glutamine for 90 min, then collected for liquid chromatography-mass spectrometry. Metabolite levels are expressed as fractional saturation of relevant ^13^C label over the total metabolite detected. *F*, glucose and glutamine uptake was measured from conditioned media samples from undifferentiated or day 15 osteogenic cultures, and day 15 levels were expressed as percent change from day 0 levels. Data are mean ± SD (n = 3 biological replicates in *A–E* and n = 4 in *F*). ∗*p* < 0.05; ∗∗∗∗*p* < 0.0001. BMSCs, bone marrow stromal (a.k.a. mesenchymal stem) cells; hBMSCs, human BMSCs.

^13^C_6_-glucose tracing of glycolytic intermediates reveals that in both undifferentiated BMSCs and throughout OB differentiation, glucose-derived labels are seen abundantly in early glycolytic metabolites such as glucose-6-phosphate (G6P) and fructose 1,6-bisphosphate (Fig. 2*A*). 1,3-diphoshoglycerate, however, sees more moderate glucose-derived labeling in undifferentiated cells which declines significantly as cells progress further down the osteogenic lineage, and both pyruvate and lactate show minimal glucose-derived carbons at any time point of the experiment. Early metabolites from the PPP also show high levels of glucose-derived labels such as ribose-5-phosphate and sedoheptulose-7-phosphate; distinct from the glycolytic intermediates, however, is that all later PPP intermediates maintain roughly 50% or greater of glucose-derived labeling throughout all phases of differentiation (Fig. 2*B*). In contrast, TCA cycle metabolites show very low levels of glucose-derived labeling in any metabolite analyzed (Fig. 2*C*). Collectively, glucose-tracing experiments indicated that in osteogenic lineage cells, glucose is metabolized primarily through early glycolytic steps and the PPP but not late glycolytic steps or TCA cycle.

The above data suggest that fuels other than glucose feed into both late steps of glycolysis and the TCA cycle. To test if glutamine is such a fuel, we performed ^13^C_5_-glutamine tracing in undifferentiated BMSCs and in cells osteoinduced for 15 days. Glutamine tracing of glycolytic metabolites shows low labeling of metabolites midway through the glycolytic pathway; however, glutamine-derived labels are present at nearly 100% fractional saturation at the late steps of the glycolytic pathway, in pyruvate and lactate (Fig. 2*D*). Similarly, TCA cycle metabolites throughout the pathway show abundant glutamine-derived labeling in both undifferentiated BMSCs and in mature OBs (Fig. 2*E*). Finally, glucose and glutamine uptake was measured at day 0 and day 15 of differentiation, and there was a dramatic shift toward glutamine uptake in OBs relative to glucose uptake (Fig. 2*F*). These data indicate that in osteolineage cells, glutamine supplies the whole TCA cycle and also late steps of glycolysis likely *via* the MAS.

### Pharmacologic inhibition of glucose-6-phosphate dehydrogenase can modulate PPP usage and osteogenic differentiation in BMSCs

**Figure 3:**
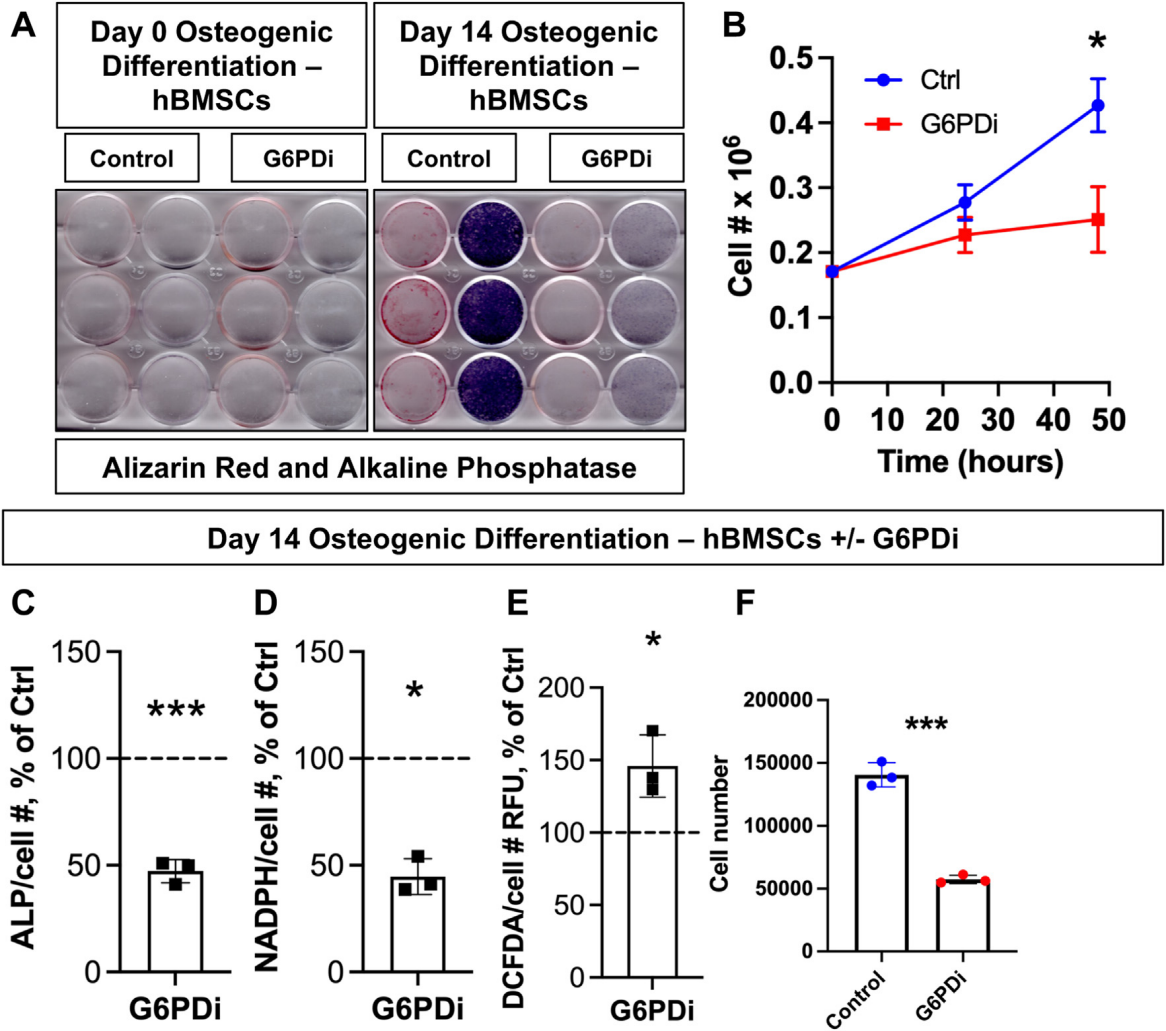
**Pharmacologic inhibition of glucose-6-phosphate dehydrogenase results in blunting of osteogenic differentiation of BMSCs.***A* and *C*, hBMSCs were osteogenically differentiated for 0 or 14 days with either vehicle treatment or 20 μM G6PDi and stained for alkaline phosphatase (ALP) or Alizarin red, and mean gray value was analyzed to quantify the ALP staining normalized to cell number marked by Hoechst 33342 staining and measured by Celigo S imaging cytometer. *B* and *F*, cell proliferation was measured by Hoechst 33342 staining and cell counting using Celigo S over 48 h in the presence or absence of G6PDi without osteogenic differentiation, and cell number was also analyzed using the same method at day 14 of osteogenic differentiation. *D* and *E*, NADPH concentration and cytosolic reactive oxygen species levels as marked by DCFDA staining were both measured using a plate reader and normalized to cell number measured in the same method as in (*B*) at day 14 of osteogenic differentiation. Data are mean ± SD (n = 3 biological replicates), unpaired *t* tests were used to determine *p* value. ∗*p* < 0.05; ∗∗∗*p* < 0.001. BMSCs, bone marrow stromal (a.k.a. mesenchymal stem) cells; G6PDi, G6PD inhibitor; hBMSCs, human BMSCs.

Given the striking data presented in Fig. 2 about the potential role of the PPP in OB maturation, we sought to further clarify the role of the PPP in the OB. BMSCs were osteogenically differentiated in the presence of G6PD inhibitor (G6PDi), a pharmacologic inhibitor of G6PD that is critical for initiation of the PPP. While undifferentiated cells treated with G6PDi showed no difference in ALP or alizarin red deposition, osteolineage cells at 14 days of differentiation showed a strong reduction in both ALP and alizarin red staining relative to control cells (Fig. 3*A*). In addition, proliferation appears to be impacted, as cell number increased at a significantly slower rate over 48 h in the G6PDi-treated group relative to controls (Fig. 3*B*), while no significant cell death was observed (not shown). Focusing on day 14 of osteogenic differentiation, G6PDi-treated cells show significantly reduced ALP staining as well as a reduction in NADPH concentration relative to vehicle-treated cells (Fig. 3, *C* and *D*). G6PDi-treated osteolineage cells also showed enhanced cytosolic reactive oxygen species (ROS) levels relative to controls, as measured by DCFDA staining, and continue to display defects in cell proliferation once differentiated as they have a reduced cell number per well relative to vehicle-treated samples (Fig. 3, *E* and *F*). Therefore, PPP plays an important role during OB differentiation, regulating proliferation, REDOX balance, and extracellular matrix biosynthesis and mineralization.

### Inhibition of the malate aspartate shuttle is detrimental to maturation osteolineage cells

**Figure 4:**
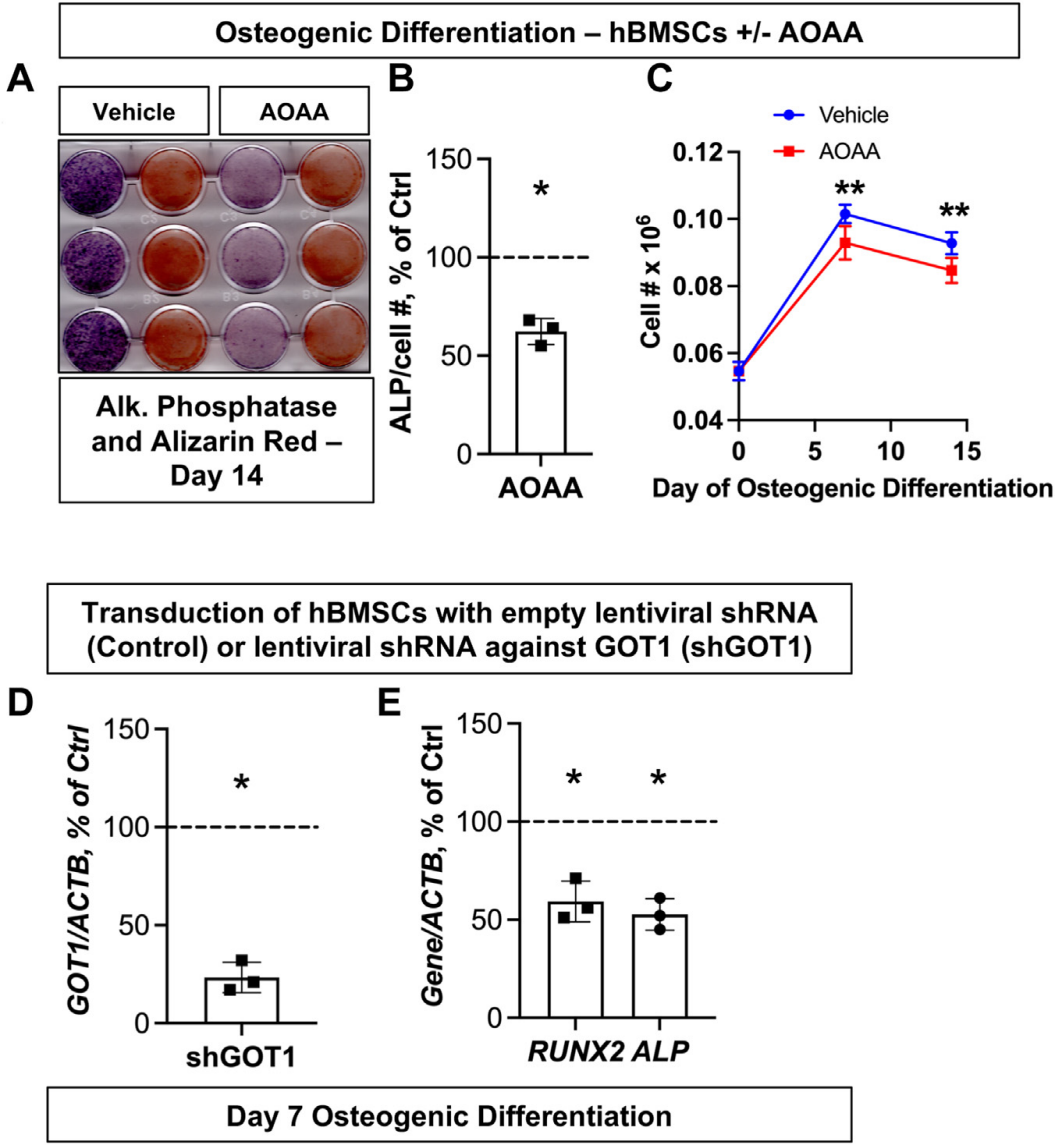
**Malate-aspartate shuttle inhibition can also reduce osteogenic differentiation in BMSCs.***A* and *B*, hBMSCs were osteogenically differentiated for 0 or 14 days with either vehicle treatment or 1 mM aminooxyacetic acid (AOAA) and stained for alkaline phosphatase (ALP) or Alizarin red, and mean gray value was analyzed to quantify the ALP staining normalized to cell number as described in Fig. 3. *C*, cell number was also analyzed at day 0 prior to treatment and at day 7 and 14 in both vehicle and AOAA-treated cells. *D* and *E*, GOT1 knockdown was obtained using lentiviral shRNA and GOT1, and osteogenic gene expression was measured using real time RT-qPCR following 7 days of culture in osteogenic media; gene of interest expression level was normalized to β-actin expression and day 0 samples to calculate fold change. Data are mean ± SD (n = 3, except in C where N = 6 biological replicates). ∗*p* < 0.05; ∗∗*p* < 0.01. BMSCs, bone marrow stromal (a.k.a. mesenchymal stem) cells; hBMSCs, human BMSCs.

Given the previous data showing the flow of glutamine-derived label toward late steps of glycolysis likely *via* MAS, we wondered if inhibition of MAS could have a blunting effect on osteogenic maturation of BMSCs. MAS may be the pathway that works synergistically with glutamine metabolism in the TCA cycle and high PPP usage in the cytosol to maintain a reducing environment in the cytosol and shuttle NADH and NAD+ across the mitochondrial membrane. To test this hypothesis, osteoinduced BMSCs were treated with aminooxyacetic acid (AOAA), an inhibitor of aminotransferase activity of the enzymes GOT1 and GOT2 which facilitate MAS activity. This resulted in a significant reduction in ALP production in AOAA-treated cells at day 14 of osteogenic differentiation, as well as a reduction in cell number relative to control-treated cells at both day 7 and day 14 (Fig. 4, *A*–*C*). In addition, we used a molecular approach to reduce MAS activity, where hBMSCs were infected with lentivirus carrying shRNA against GOT1 or scrambled lentiviral shRNA as a control. GOT1 shRNA-infected cells showed reduced expression of *GOT1*, as well as a reduction in *RUNX2* and *ALP* osteogenic markers during osteogenic differentiation and maturation (Fig. 4, *D* and *E*). Therefore, MAS is required for osteogenic differentiation likely by working synergistically with the glucose-fed PPP and glutamine-fed TCA cycle.

## Discussion

While the genetic and intracellular signaling pathways governing BMSC differentiation down the osteogenic lineage have been well described in previous studies, the energetic demands of these cells during this process are less clear. In this study, we sought to clarify the metabolic flux through the process of osteogenic differentiation of hBMSCs into OBs by utilizing ^13^C_6_-glucose or ^13^C_5_-glutamine to trace glucose and glutamine usage, respectively. These data have shown that glucose primarily contributes to early glycolytic intermediates but then preferentially shunts toward entry into the PPP rather than continuing through the later stages of glycolysis and TCA cycle. This metabolic setup has not been yet described in osteolineage cells but only in some cancer cells (24, 25, 26). This PPP preference, however, allows for production of NADPH that maintains the reducing environment within the cytosol needed to allow for redox reactions and biosynthesis of nucleotide precursors used to power proliferation, as well as production of extracellular matrix proteins. The reduced flow of glucose into late glycolytic steps in osteolineage cells has been noticed before; however, the potential causes of this phenomenon have not been explored (6, 7).

In order for the PPP to dominate the use of glucose in osteolineage cells at the expense of late glycolytic and TCA cycle intermediates, the latter have to be supplied from other sources, such as glutamine. Glutamine is primarily metabolized in mitochondria feeding into the TCA cycle and potentially into late steps of glycolysis *via* the MAS, and this preferential usage of glutamine was seen regardless of time point during osteogenic differentiation. We in fact observed that to provide for this enhanced demand, glutamine is taken up at a greatly increased level relative to glucose in osteogenically differentiated cells, a finding that has been confirmed by other groups (3, 27, 28). While the uptake increased at day 15, there was no change in glutamine metabolic tracing from day 0, likely because the glutamine contribution was already maximized prior to differentiation. Due to this increased level of uptake of glutamine at day 15, it is likely that both glutamine uptake and subsequent labeling of metabolic intermediates of the TCA cycle and late glycolysis happened more quickly in osteoinduced cells than at day 0, but we measured all labeling at 90 min in order to compare them. This also aligns with the findings that the glutamine transporter SLC1A5 is critical for regulation of OB differentiation and protein synthesis (29). Glutamine-derived labeling was also observed in late glycolytic intermediates suggesting the role of MAS in this process. High MAS activity in OBs was in fact reported before (9). These findings give additional important context to previous findings describing a glycolytic shift during the process of aging in bone (13, 23), based on the reliance on the PPP in BMSCs and OBs during bone homeostasis.

Inhibition of G6PD pharmacologically in hBMSCs during the osteogenic maturation process resulted in a reduction in NADPH production and cell proliferation, as well as an increase in cytosolic ROS levels. As a result, a strong reduction of ALP deposition per cell was seen. This result was also replicated in ST2 cells, a mouse mesenchymal stem cell line (Fig. S1). This effect was replicated, albeit to a lower level, with use of AOAA or using genetic knockdown of GOT1 to inhibit the MAS, which is responsible for providing transport of NAD+ and NADH between the cytosol and the mitochondria to maintain a reduced cytosolic environment. Therefore, MAS works in concert with the glucose-driven PPP and the glutamine-driven TCA cycle in BMSCs that have begun to differentiate into OBs. Importantly, we also saw a reduction in lipid accumulation (lipid-specific Nile Red staining) and cell proliferation during adipogenic differentiation of BMSCs treated with G6PDi. Therefore, the effect of inhibition of PPP may not be specific to osteogenic differentiation, though G6PDi effect on osteogenic differentiation was more robust relative to adipogenic differentiation, suggesting higher dependence of osteogenic lineage on PPP (Fig. S1).

In summary, we here report a novel finding of unusual metabolic setup in osteolineage cells, *i.e.*, preferential flux of glucose-derived intermediates into the PPP and flux of glutamine-derived intermediates into both TCA cycle and late steps of glycolysis. Such a setup may be beneficial for these cells as they have high capacity for both rapid proliferation and robust matrix biosynthesis, *e.g.*, during bone fracture. Improved understanding of the metabolic flux within osteolineage cells delineated by this study may be leveraged into novel therapeutic avenues to enhance the bone formation capabilities by OBs during fracture repair or the aging process.

## Experimental procedures

### Cell culture, osteoinduction, and inhibitor treatments

hBMSCs were purchased from Lonza and cultured in Dulbecco's modified Eagle's medium containing 5 mM glucose, 1 mM glutamine, 10% FBS containing adequate amount of fatty acids, and no pyruvate. Cells were cultured in a 5% CO_2_ cell culture incubator with low (physiological) O_2_ (5%). At confluency, hBMSCs were osteogenically induced with 50 μg/ml ascorbate (TCI America, TCI A2521) and 2.5 mM β-glycerophosphate (USB, #216155) for 0, 5, 10, 14, or 15 days. Experiments using G6PD inhibitor, G6PDi (Cayman Chemicals, #31484), were performed using a concentration of 20 μM. To inhibit the malate-aspartate shuttle, cells were treated with AOAA at 1 mM (Cayman Chemicals, #28298). For all osteogenic differentiation experiments, inhibitor treatments were initiated at start of osteoinduction and during all subsequent media changes. For experiments involving evaluation of proliferation using inhibitors during 48 h of culture, no osteoinduction media were used.

### ^13^C glucose and glutamine labeling and liquid chromatography mass spectrometry

Following osteoinduction to the relevant time point, cells were labeled with either 5 mM ^13^C_6_-glucose or 1 mM ^13^C_5_-glutamine for 90 min. Cells were scraped on dry ice in 1 ml of dry ice-cold 80% MeOH (Omnisolv) to extract metabolites; samples were then vortexed and spun at 12,000 RPM for 5 min, the supernatant was saved, and the pellet was re-extracted using 0.5 ml of additional 80% MeOH. Samples were then dried under nitrogen stream and reconstituted in 150 μl of 50% MeOH. Samples were analyzed using reverse-phase liquid chromatography with an ion-pairing reagent in a Shimadzu HPLC (Shimadzu Scientific Instruments) coupled to a Thermo Quantum triplequad mass spectrometer (MS, Fisher Scientific).^13^C labeling was determined as ‘Fractional saturation’, *i.e.*, ^13^C-labeled/total.

### Glucose and glutamine uptake assay

Cells were induced to differentiate for indicated times or left untreated. To measure glucose and glutamine consumption, media samples from cells were taken 24 h apart and ran on a Bioprofile Flex analyzer (Nova Biomedical) to calculate consumption. Data were normalized to the cell number obtained by staining with Hoechst 33342 (Molecular Probes H1339) and quantifying with Celigo S imaging cytometer (Nexcelcom Bioscience).

### ALP staining and activity assay, alizarin red staining, and cell number quantification

OB differentiation was assessed by ALP staining using NBT/BCIP 1-step (Fisher Scientific, 34,042) and alizarin red staining (Sigma, A5533) and quantified using ImageJ as previously described (30). ALP activity assay was performed using the AttoPhos fluorescent substrate system per manufacturer’s instructions (Promega, S1000). ALP staining signal was normalized to cell number obtained with DAPI staining (Fisher Scientific, D1306) quantified using Celigo S counter. ALP activity was normalized to protein content measured by BCA assay (Fisher Scientific, #23277). Cell number counts were performed using Hoechst 33342 stain and Celigo S counter.

### NADPH quantitation assay

Cells plated in 6-well plates were scraped and harvested on day 14 of osteogenic differentiation in 150 μl of NADP/NADPH extraction buffer, and samples were prepared per manufacturer’s instructions for the NADP/NADPH quantification colorimetric kit (Biovision, #K347). Prior to harvest, cells were stained with Hoechst 33342 and counted using Celigo S counter. Samples were read on a Biotek Synergy H1 microplate reader at *A*_450_ after 4 h; NADPH concentration was normalized to cell number.

### DCFDA staining

Cytosolic ROS was measured using DCFDA staining in a 96-well plate. Prior to harvest, cells were stained with Hoechst 33342 and counted using Celigo S counter. Cells were then incubated with 1 μM DCFDA (Fisher Scientific, D399) for 30 min at 37 °C and washed following incubation. Fluorescent signal was then measured on a Biotek Synergy H1 microplate reader at 488/530 nm (Ex/Emm), and the signal was normalized to cell number.

### Real-time reverse transcriptase polymerase chain reaction

Total RNA was isolated using the RNeasy kit (Qiagen, 74,106) and reverse transcribed into cDNA using the qScript cDNA synthesis kit (Quanta, 95,048–500). Real-time reverse transcriptase polymerase chain reaction was performed in the RotorGene system (Qiagen) using SYBR Green (Quanta, 95,072–012). The expression of genes of interest was normalized to the expression of *ACTB* gene and expressed as a fold change relative to day 0. The following primer pairs were used: human *RUNX2* (5′-TCC GGA ATG CCT CTG CTG TTA TGA-3′ and 5′-ACT GAG GCG GTC AGA GAA CAA ACT-3′), *ALP* (5′-TGC AGT ACG AGC TGA ACA GGA ACA-3′ and 5′-TCC ACC AAA TGT GAA GAC GTG GGA-3′), *BGLAP* (5′-CCCT CACA CTCC TCGC CCTATT-3′ and 5′-ATAG GCCT CCTG AAAG CCGATGT-3′), and *ACTB* (5′-AGC CAT GTA CGT TGC TAT CC -3′ and 5′-CGT AGC ACA GCT TCT CCT TAA T-3′).

### Lentiviral shRNA induction

GOT1 knockdown was done using lentiviral shRNA. Cells were plated at 10^5^/well in 12-well plates and infected with GOT1 shRNA lentiviral particles (Sigma, pLKO.1-puro backbone, clone ID TRCN0000034785) or control empty vector particles (Sigma, # SHC016V) at 5 MOI of shRNA or control vector with addition of Polybrene (Sigma, #TR-1003) at 8 mg/ml. Plates were spun in a bucket centrifuge at 1000 rpm for 20 min to force viral particles on cell layer. After 48 h, puromycin at 10 mg/ml was added to achieve stable knockdown of GOT1. Following an additional 48 h, stably transduced cells were then used for osteogenic differentiation experiments with a maintenance dose of puromycin at 2 mg/ml included in the media.

## Data availability

All data generated or analyzed during this study are included in the manuscript and supporting files. All the raw data and images are available upon request.

## Supporting information

This article contains supporting information.

## Conflict of interest

The authors declare that they have no conflicts of interest with the contents of this article.
