# Supplementary material for: Energy metabolism in osteoprogenitors and osteoblasts: Role of the pentose phosphate pathway
Source: J Biol Chem. 2024 Nov 26;301(1):108016. doi: 10.1016/j.jbc.2024.108016 (PMC11721538; doi:10.1016/j.jbc.2024.108016)

**Energy metabolism in osteoprogenitors and osteoblasts:**

**role of the Pentose Phosphate Pathway – Supporting Information**

##### ^a^Sarah E. Catheline, ^a^Charles O. Smith, ^a^Matthew McArthur, ^a^Chen Yu, ^b,c,d^Paul S. Brookes, ^a,c,d^Roman A. Eliseev.

##### ^a^Center for Musculoskeletal Research, University of Rochester, Rochester, NY 14624, USA

##### ^b^Department of Anesthesiology and Perioperative Medicine, University of Rochester, Rochester, NY, 14624, USA.

##### ^c^Department of Pharmacology & Physiology, University of Rochester, Rochester, NY 14624, USA

##### ^d^Department of Pathology, University of Rochester, Rochester, NY 14624, USA

Supporting information – Methods, Figure and Figure Legend

**ST2 cell culture, osteogenic induction, and adipogenic induction**

Mouse long bone-derived ST2 cells were a gift from Dr Clifford Rosen (Maine Medical Center). Cells were maintained in a 37°C incubator at 5% CO_2_ in low glucose αMEM media (Gibco A10490-01) containing 1 mM L-glutamine, ribonucleosides (0.01 g/L each), deoxyribonucleosides (0.01 g/L each), no ascorbic acid, 10% FBS, and 1% pennicillin/streptomycin (Gibco 15140-122). For osteogenic differentiation, cells were induced with 50 μg/ml ascorbate and 2.5 mM β-glycerophosphate for 14 days. For adipogenic differentiation, cells were induced in high-glucose DMEM (Gibco, 25 mM glucose) supplied with 1 μM dexamethasone, 0.5 mM IBMX, 1 μM rosiglitazone, and 10 μg/ml insulin for 2 days and with rosiglitazone and insulin for the remaining 5 days. Media were changed every 3 days. Adipogenic differentiation was assessed by Nile Red staining and normalized to cell number assessed via nuclear Hoechst 33342 staining and Celigo S imaging cytometer (Nexcelcom Bioscience).

**Figure S1. Pharmacologic inhibition of glucose-6-phosphate dehydrogenase results in reduced osteogenic and adipogenic differentiation of mouse BMSCs.** ST2 were osteogenically differentiated for 14 days with either vehicle treatment or 20 μM G6PDi and stained for alkaline phosphatase (ALP) or Alizarin red (**A**) and mean gray value was analyzed to quantify the ALP staining (**B**) normalized to cell number marked by Hoechst 33342 staining and measured by Celigo S imaging cytometer. ST2 were adipogenically differentiated for 7 days with either vehicle treatment or 20 μM G6PDi and stained for Nile red (**D, E**), staining intensity was measured by Celigo S and normalized to cell number marked by Hoechst 33342 staining. Cell number was also analyzed using the same method at day 14 of osteogenic differentiation or day 7 of adipogenic differentiation (**C, F**). Data are mean ± SD (n = 3 biological replicates, except Nile red staining where N = 6 biological replicates).


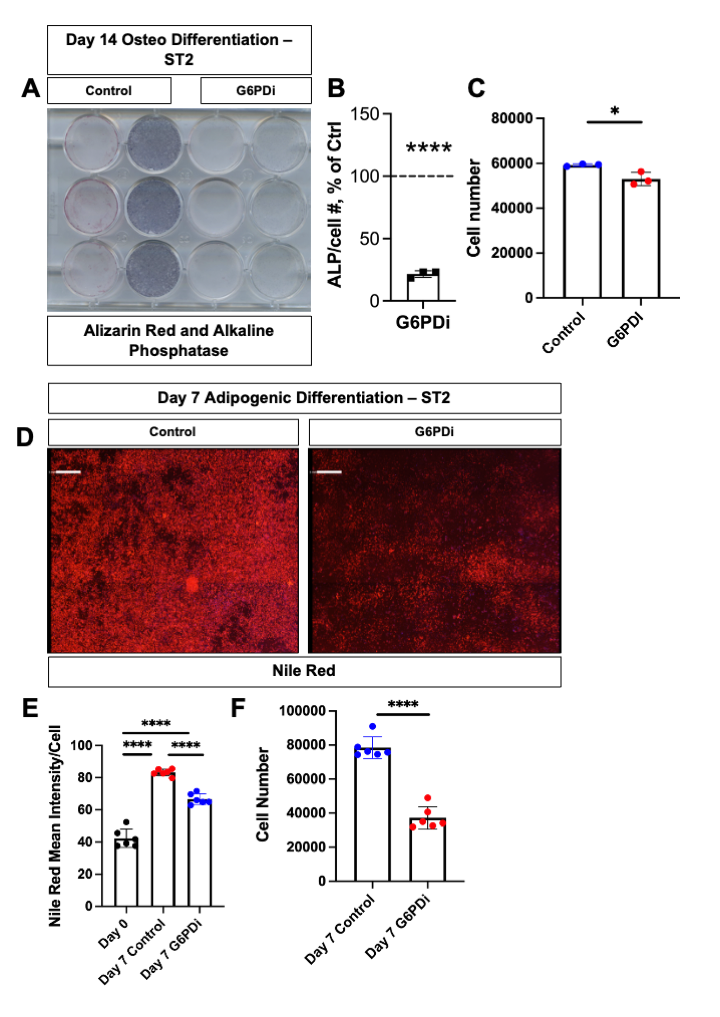

Supplement: Supporting Information [file mmc1.docx]
